# Seasonal Dynamics of Haptophytes and dsDNA Algal Viruses Suggest Complex Virus-Host Relationship

**DOI:** 10.3390/v9040084

**Published:** 2017-04-20

**Authors:** Torill Vik Johannessen, Aud Larsen, Gunnar Bratbak, António Pagarete, Bente Edvardsen, Elianne D. Egge, Ruth-Anne Sandaa

**Affiliations:** 1 Vaxxinova Norway AS, Kong Christian Frederiks plass 3, 5006 Bergen, Norway; torillvjohannessen@gmail.com; 2 Uni Research Environment, N-5008 Bergen, Norway; aud.larsen@bio.uib.no; 3 Department of Biology, University of Bergen, N-5020 Bergen, Norway; gunnar.bratbak@bio.uib.no (G.B.); antonio.pagarete@uib.no (A.P.); 4 Department of Biosciences, University of Oslo, 0316 Oslo, Norway; bente.edvardsen@ibv.uio.no (B.E.); elianne.egge@gmail.com (E.D.E.)

**Keywords:** Haptophyta, *Phycodnaviridae*, Megaviridae, viral–host interactions, metagenomics, marine viral ecology

## Abstract

Viruses influence the ecology and diversity of phytoplankton in the ocean. Most studies of phytoplankton host–virus interactions have focused on bloom-forming species like *Emiliania huxleyi* or *Phaeocystis* spp. The role of viruses infecting phytoplankton that do not form conspicuous blooms have received less attention. Here we explore the dynamics of phytoplankton and algal viruses over several sequential seasons, with a focus on the ubiquitous and diverse phytoplankton division Haptophyta, and their double-stranded DNA viruses, potentially with the capacity to infect the haptophytes. Viral and phytoplankton abundance and diversity showed recurrent seasonal changes, mainly explained by hydrographic conditions. By 454 tag-sequencing we revealed 93 unique haptophyte operational taxonomic units (OTUs), with seasonal changes in abundance. Sixty-one unique viral OTUs, representing Megaviridae and *Phycodnaviridae*, showed only distant relationship with currently isolated algal viruses. Haptophyte and virus community composition and diversity varied substantially throughout the year, but in an uncoordinated manner. A minority of the viral OTUs were highly abundant at specific time-points, indicating a boom-bust relationship with their host. Most of the viral OTUs were very persistent, which may represent viruses that coexist with their hosts, or able to exploit several host species.

## 1. Introduction

Marine phytoplankton account for approximately 50% of global primary production and have a strong impact on global nutrient cycling [1]. As key components within the phytoplankton community in both coastal and open oceans, and at all latitudes [2], haptophytes play important roles both as primary producers but also as mixotrophs, grazing on bacteria and protist [3]. Blooms of haptophytes can have significant ecological and economic impacts both through the amount of organic matter being produced and through production of toxins harmful to marine biota [4]. Most haptophyte species, however, do not usually form blooms, but rather appear at low concentrations at all times [5,6,7].

Phytoplankton diversity, abundance, and community composition change through the seasons, driven by variations in environmental conditions and biological processes. Viruses can, in theory, significantly condition those dynamics. Viral-based phytoplankton lysis can be at least as significant as grazing [8,9] and have the potential to drastically change host community structure [10]. Viral activity related to bloom forming haptophytes like *Emiliania huxleyi*, *Phaeocystis pouchetii*, and *Phaeocystis globosa* has been well studied [9,11,12,13,14]. During such blooms, viruses exhibit a strong regulatory role, and contribute to the termination of the bloom in what may be referred to as a “boom and bust” relationship [11,15,16]. Viruses may also prevent bloom formation by keeping host population at non-blooming levels [16,17,18]. Such interactions between host and virus have been described as a stable coexistence and explained by viral resistance, immunity and/or strain specificity [17,19,20,21,22,23].

The low diversity and high abundance, which characterize phytoplankton blooms, give species of specific viruses ample possibilities to find susceptible hosts. Most haptophyte species, such as species belonging to the Prymnesiales, however, are part of highly-diverse communities and occur at low concentrations [5,6,7], which decrease their chance of being infected by viruses with specific host requirements. Nevertheless, viruses infecting both *Prymnesium* and *Haptolina* species (order Prymnesiales) have been isolated, but have several characteristics that distinguish them from viruses infecting bloom-forming haptophytes like *E. huxleyi* [24,25]. Studies describing the seasonal diversity and abundances of these viruses and their potential host communities (haptophytes) in the environment are scarce.

All known haptophyte viruses have double-stranded DNA (dsDNA) genomes and belong to two related viral families, the *Phycodnaviridae* and Megaviridae, within a monophyletic group of nucleocytoplasmic large DNA viruses (NCLDV) [26]. Phycodnaviruses infect prasinophytes, chlorophytes, raphidophytes, phaeophytes, and haptophytes [27]. The Megaviridae family, not yet recognized as a taxon by the International Committee on Taxonomy of Viruses (ICTV), consists of NCLDVs infecting both non-photosynthetic protists such as *Acanthamoeba* and *Cafeteria roenbergensis* [28,29], as well as photosynthetic ones including prasinophytes, pelagophytes and prymnesiophytes (haptophytes) [14,30,31]. Both *Phycodnaviridae* and Megaviridae are abundant in aquatic environments but the majority are uncultured and not yet described [31,32,33,34,35,36,37]. The diversity within these two families is high, and available primers only match a fraction of its representatives [32,38,39]. Moreover, only few polymerase chain reaction (PCR) primer-sets that target *Phycodnaviridae* and Megaviridae families are currently available [32,38]. The DNA polymerase B primers (polB) capture a wide diversity within the *Phycodnaviridae* family including the prasinoviruses and chloroviruses [36,37,38,39] whereas the major capsid protein (MCP) primers are better suited for capturing the diversity of the Megaviridae family including prymnesioviruses that infect various haptophytes ([32,39], this study). Coccolithoviruses (e.g., *Emiliania huxleyi virus* (EhV)), a diverged group in the *Phycodnaviridae* family, are not targeted by any of these primer-sets.

In previous studies, we have described the microbial community dynamics of the seasonal spring- and fall-blooms in a West Norwegian open fjord system (Raunefjorden) [40,41]. Virus infection seems to be one of the factors that drive the succession in the haptophyte community after the typical diatom spring bloom [40]. In the present study, we follow up on these investigations using methods with higher taxonomic resolution that enable a more specific focus on haptophytes and their potential viruses. By following dynamics and diversity in virus and haptophyte communities over a two-year period, we aimed at revealing the possible regulatory role of viruses, not only during blooms but also during periods with higher community diversity and lower productivity such as late fall and winter.

## 2. Results

### 2.1. Microbial Abundance and Abiotic Factors

**Figure 1:**
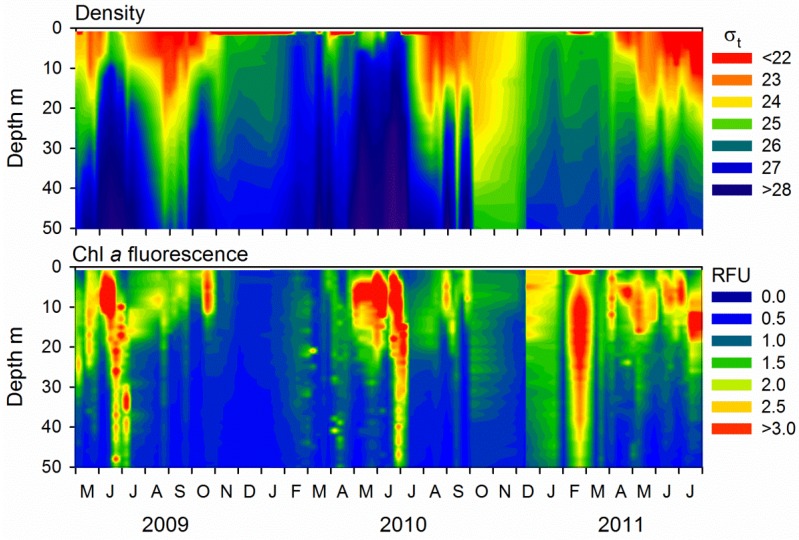
Isopleth diagrams showing seawater density (σt) and chlorophyll *a* (Chl *a*) fluorescence (RFU = relative fluorescence units) at the sampling station in Raunefjorden, respectively.

The phytoplankton spring bloom, identified as elevated chlorophyll *a* (Chl *a*) fluorescence, started in late February before any stratification of the water masses, and lasted longer in 2011 than in 2010 (Figure 1). The water masses in Raunefjorden started to stratify in March–April, and the stratification was more pronounced and deeper in 2011 than in 2010 (Figure 1).

Several minor upwelling events and exchange of water masses were evident in spring (e.g., in June 2009, April 2010 and June 2010); concurrently May and June were characterized by several successive blooms with high Chl *a* levels (2–8 µg per L). The pycnocline deepened throughout summer and fall before the seasonal inflow and upwelling caused deep mixing in late fall, which corresponded to a temporary, slight increase in Chl *a* concentrations in fall each year (October–November). The water masses were well mixed through fall and winter.

**Figure 2:**
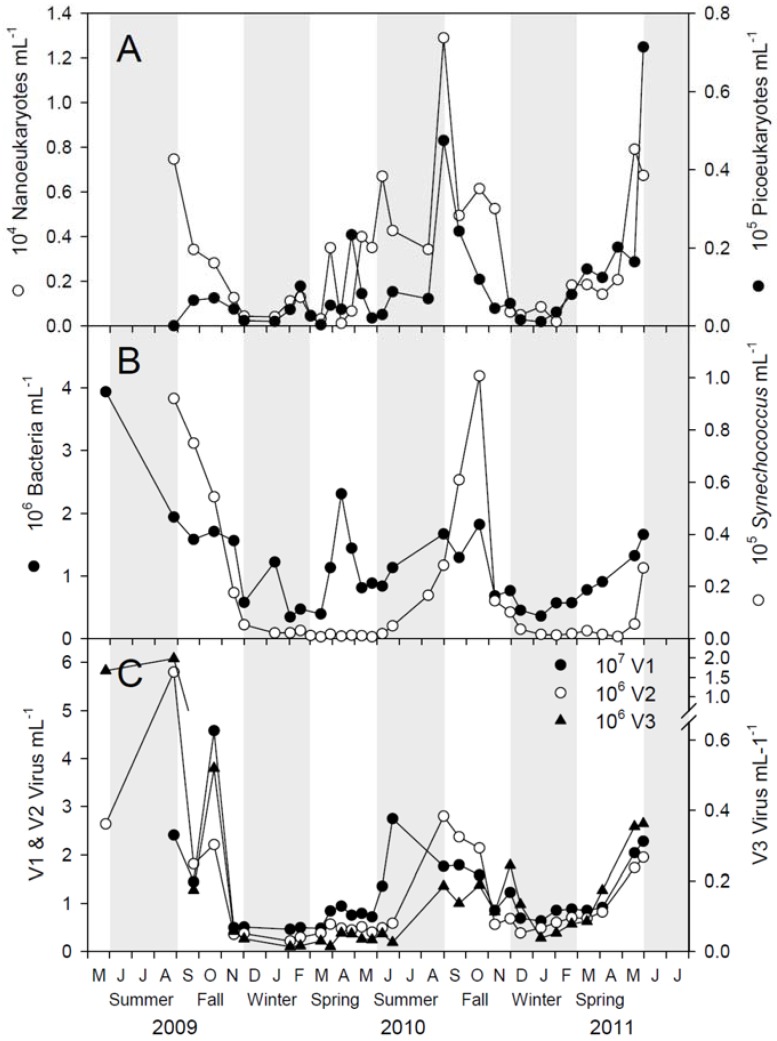
Abundance of microbial plankton at the sampling station in Raunefjorden measured by flow cytometry. (**A**) Phototrophic picoeukaryotes (filled circles) and nanoeukaryotes (open circles); (**B**) *Synechococcus* (open circles) and total bacteria (filled circles); and (**C**) V1 (low fluorescence viruses), V2 (intermediate fluorescence viruses) and V3 (high fluorescence viruses).

The first increase in pico- and nano-eukaryote abundance, as measured by flow cytometry, was observed in late February (Figure 2A). The cell numbers increased throughout spring and summer with maximum abundance of both groups in August 2010 and May/June 2011. Total bacterial abundance was variable with a decreasing trend in fall and winter and an increasing trend in spring and summer-fall (Figure 2B), while the *Synechococcus* (cyanobacteria) abundance peaked once each year in late summer-fall (Figure 2B).

Viral abundance increased in the spring and summer. The highest values were found during summer and fall. The abundance of all three viral groups varied in synchrony (V2 vs. V1: *r* = 0.603, df = 26, *p* < 0.0007, V3 vs. V1: *r* = 0.483, df = 26, *p* < 0.0091) and the smaller viruses (V1) outnumbered the larger viruses (V2 and V3) by a factor of approximately 5–20 and 50–300, respectively (Figure 2C). Correlation analysis showed that the viral abundance was correlated (*p* < 0.01) with the abundance of bacteria, cryptophytes and *Synechococcus* (Table S1). The abundance of small-sized viruses (V1) also correlated with Chl *a* and abundance of nanoeukaryotes, while the abundance of intermediate-sized viruses (V2) correlated with abundance of picoeukaryotes and nanoeukaryotes.

### 2.2. Haptophytes

**Figure 3:**
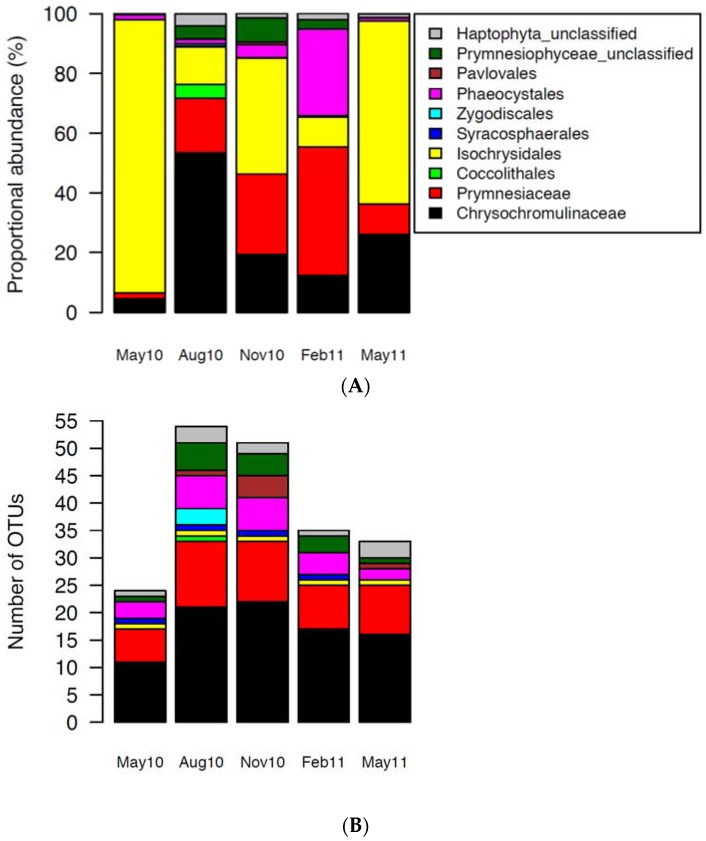
(**A**) Relative abundance of operational taxonomic units (OTUs) in the different haptophyte orders or clades. The OTUs represented seven accepted haptophyte orders: Pavlovales, Phaeocystales, Coccolithales, Isochrysidales, Syracosphaerales, Zygodiscales and Prymnesiales. The latter is represented by the families Chysochromulinaceae and Prymnesiaceae. The reads that did not belong to any of these orders were assigned to class Prymnesiophyceae (unclassified) or Haptophyta (unclassified); (**B**) Number of unique OTUs (richness) in each sample after rarifying to lowest read abundance (i.e., subsampling to obtain equal sample size).

Haptophyte reads (sequences), clustered based on 98% nucleotide sequence similarity, formed a total of 93 operational taxonomic units (OTUs) (Table S2). OTUs were classified against a curated Haptophyta reference sequence database [42] to the lowest reliable taxonomic level (Table S2). The classified OTUs were placed into one of seven haptophyte orders: Pavlovales, Phaeocystales, Zygodiscales, Syracosphaerales, Isochrysidales, Coccolithales and Prymnesiales (Figure 3). A number of the reads could not be classified to these formally-accepted taxa, and were assigned to defined clades without cultured representatives according to [42], here named Haptophyta unclassified (Clades HAP2, HAP3, HAP4, and HAP5) and Prymnesiophyceae unclassified (Clades B3, B4, D, E and F) (Table S2). Prymnesiales is, in Figure 3, divided into the families Chysochromulinaceae and Prymnesiaceae. OTUs assigned to the order Isochrysidales all belonged to the *E. huxleyi* cluster. A more detailed classification of the 93 haptophyte OTUs to is shown in Table S2.

**Figure 4:**
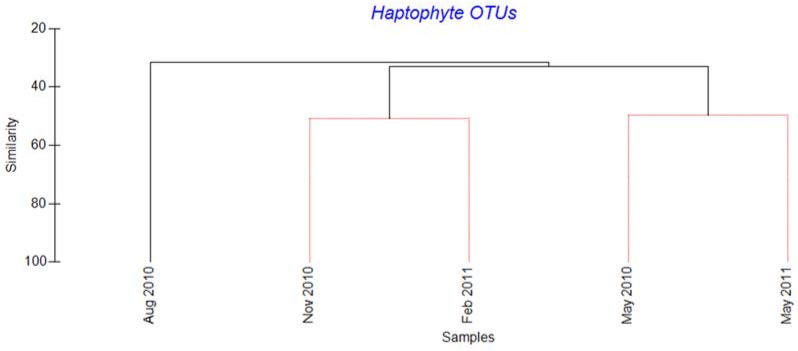
Cluster dendrogram illustrating Bray-Curtis dissimilarity in the haptophyte OTU compositions between the five samples from Raunefjorden. OTUs were defined as reads with ≥98% nucleotide similarity. Sequences were normalized to 100 in each sample and log-transformed prior to similarity calculations. Samples connected by red lines were not significantly differentiated (SIMPER permutation test). Black lines indicate significant differentiation (*p* < 0.05, SIMPER permutation test).

Diversity and community composition varied between the samples, with highest diversity in August and lowest in May (Shannon diversity values of 2.97 and 0.50 respectively, Table S3). Based on the Bray-Curtis dissimilarity analysis we found that the August 2010 sample differed most from the rest (Figure 4).

OTUs assigned to Isochrysidales (only *E. huxleyi*) were present in all samples, with particularly high relative abundance in May. OTUs assigned to Prymnesiales, i.e., the families Prymnesiaceae and Chrysochromulinaceae, dominated the samples from August, November and February, while OTUs belonging to Phaeocystales occurred in high relative abundance only in February. Diversity was highest within Prymnesiales, with 35 and 21 unique OTUs assigned to Chrysochromulinaceae and Prymnesiaceae, respectively (Figure 3B). Ten different haptophyte OTUs were present in all samples, one was classified to *E. huxleyi*, one to Clade F, and eight to Prymnesiales (Table S2).

### 2.3. Megaviridae and Phycodnaviridae

**Figure 5:**
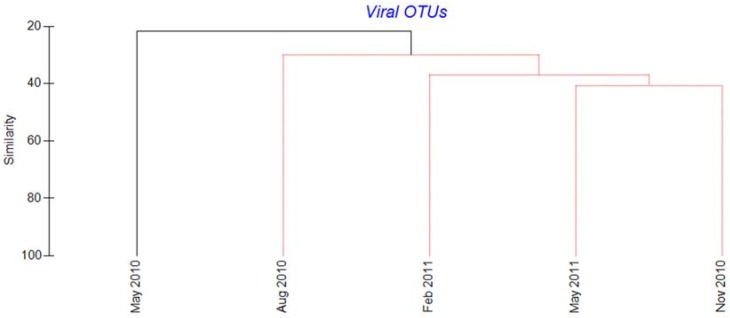
Cluster dendrogram illustrating Bray-Curtis dissimilarity between the virus OTU compositions in five samples from Raunefjorden. OTUs were defined as sequences with >95% amino acid similarity. The sequence data were normalized to 100 in each sample and log-transformed prior to similarity calculations. Samples connected by red lines could not be significantly differentiated (SIMPER permutation test). Black lines indicate significant differentiation (*p* < 0.05, SIMPER permutation test).

All the quality-trimmed viral reads showed similarity to algal viruses in the Megaviridae and *Phycodnaviridae* families, with BLAST scores between 50 and 90% amino acid sequence identity. OTU clustering based on 95% amino acid identity gave a total of 161 OTUs containing 10 or more sequences (Table S4), with 61 being unique (Table S5). Forty-one and 20 of these OTUs showed highest similarity to the Megaviridae and *Phycodnaviridae* families, respectively (Table S5). Half of the OTUs (53%) were rare, each comprising less than 1% of the total reads (Table S5). The diversity was highest in May 2010 and lowest in February 2011 (Shannon diversity values of 2.66 and 1.45, respectively) (Table S4). Based on Bray-Curtis dissimilarity, the May 2010 sample differed significantly from the other 4 samples (Figure 5).

**Figure 6:**
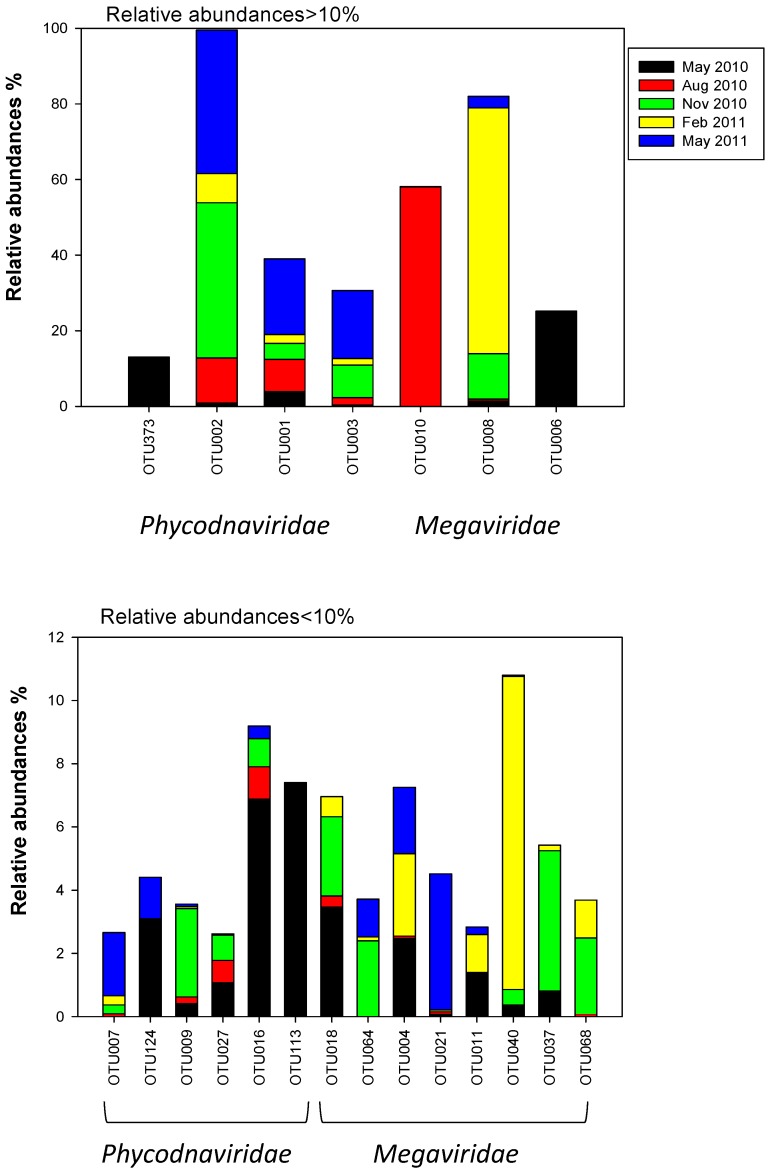
Relative abundances of 21 different OTUs containing more than 50 reads in the different samples.

Fifty-eight percent of the viral OTUs were found in more than two of the samples (Table S5). Five viral OTUs (OTU009, OTU002, OTU001, OTU003, OTU008) were present in all the samples and dominated the samples from November, February2010 and May 2011. Four of these clustered within the *Phycodnaviridae* family. Others, such as OTU006, OTU373 and OTU010 that dominated the samples taken in May and August 2010, respectively, were almost absent or undetectable the rest of the year (Table S5, Figure 6).

**Figure 7:**
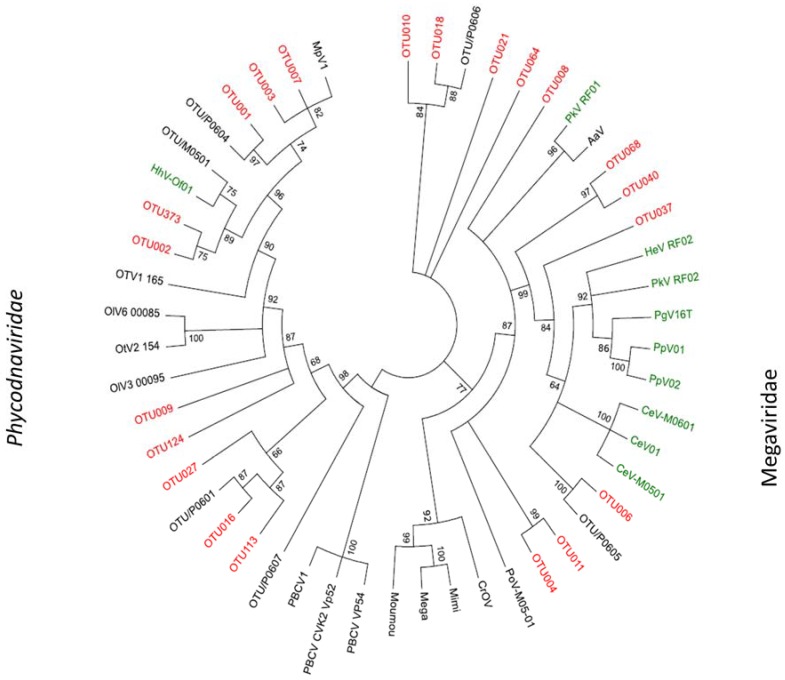
Midpoint rooted phylogenetic tree of the most abundant OTUs (>50 reads, marked in red) with similarities to the Megaviridae and *Phycodnaviridae* families, respectively. The tree was calculated based on the DNA-sequences encoding partial MCP-genes (FastTree v2.1.8 with default parameters). Bootstrap values form 100 replicates and aLRT- likelihood-values >0.5 are shown on nodes. Abbreviations: CroV; *Cafeteria roenbergensis virus*, Moumou; *Moumouvirus goulette*, Mimi; *Mimivirus,* Mega; *Megavirus chiliensis*, AaV; *Aureococcus anophagefferens virus*, PoV; *Pyramimonas orientalis virus*, PkV; *Prymnesium kappa virus*, HeV; *Haptolina ericina virus*, HhV; *Haptolina hirta virus*, CeV; *Chrysochromulina ericina virus*, PgV; *Phaeocystis globosa virus*, PpV; *Phaeocystis pouchetii virus*, PBCV; *Paramecium bursaria Chlorella virus*, MpV; *Micromonas pusilla virus*, OsV; *Ostreococcus* sp. *virus*, OlV; *Ostreococcus lucimarinus virus*. OTU/P0605, OTU/P0606, OTU/P0604, OTU/P0607, OTU/P0601, OTU/M0501 are all sequences from an earlier study in Raunefjorden [32]. Reference strains marked in green are haptophyte-infecting viruses maintained in culture.

The Megaviridae-like OTUs dominated the samples from August and February (Figure 6, Table S5). Three OTUs occurred in relative abundances over 10% (OTU010, OTU008 and OTU006). Two of them dominated the samples in August and February, the third dominated in May 2010. Further, eight OTUs occurred in low relative abundances (<10%) and were present in three or more samples. The most abundant algal Megaviridae-like OTU, OTU008, was distantly related to a virus infecting the haptophyte *Prymnesium kappa* (RF01) [25], with low bootstrap support (41%) (Figure 7). OTU008 dominated the sample from February (65%) and was present in all samples (relative abundances between 0.32–65%) (Figure 6, Table S5). The single most abundant OTU in May samples (OTU006) was also grouped within Megaviridae and clustered together with viruses infecting different haptophytes such as *Chrysochromulina, Phaeocystis* and *Prymnesium* (Figure 6 and Figure 7). Three other OTUs (OTU037, OTU068 and OTU040) clustered within this clade as well. OTU037 and OTU040 were present at relatively low abundances (0.04–9.9%) in May and November 2010, and February 2011, while OTU068 were present at relative low abundances (0.09–2.4%) in samples from August and November 2010, and February 2011 (Figure 6 and Figure 7, Table S5). Three new branches were made next to the Megaviridae family consisting of 4 OTUs from this study (Figure 7) where OTU010 and OTU18 clustered together with an environmental sequence from an earlier study at the same site [32].

OTU010 dominated in the sample from August (58%) and occurred at low relative abundances (0.04–0.05%) in the samples from November 2010 and May 2011 (Table S5). OTU18 occurred at low abundances in samples from May 2010, August, November and February. The two other collapsed branches comprised OTU064 and OTU021, both occurring at low abundances at three and four sample periods, respectively (Figure 6, Table S5). The two last OTUs in the Megaviridae family (OTU011, OTU004) showed highest similarity to a virus infecting the chlorophyte *Pyromimonas orientalis*. Both occurred at less than 10% in samples from May 2010, February 2011 and May 2011.

The *Phycodnaviridae* OTUs (Figure 6, Table S5) consisted of four OTUs (OTU373, OTU002, OTU001, OTU003) with relative abundances over 10% and six OTUs (OTU007, OTU124, OTU009, OTU027, OTU016, OTU113) with relative abundances below 10%. Three OTUs (OTU001, OTU002 and OTU003) dominated the samples from November and May 2011. They cluster within two subclades together with two cultured viruses, the prasinovirus *Micromonas pusilla virus*, and an unclassified virus shown to infect the haptophyte *Haptolina hirta* (HhV-Of01) [43] (Figure 7). Both clades also included environmental clones previously obtained at the same location [32]. The OTU016 and OTU113 also grouped together with an environmental clone from Raunefjorden [32]. OTU113 occurred only in May 2010 while OTU016 in addition were present on three other occasions (Figure 6, Table S5). OTU009, OTU124 and OTU027 did not match any viral sequences in GenBank. They were never abundant (relative abundances between 0.04–3.1%), but some were frequently observed (e.g., OTU009 and OTU027) (Figure 6, Table S5).

## 3. Discussion

### 3.1. Seasonal Patterns in Microbial Dynamics and Environmental Factors

The biological variables (Chl *a* concentration, phytoplankton, bacterial, and viral abundances) followed a seasonal and recurrent pattern that corroborated earlier descriptions of the microbial community in Raunefjorden [40,41,44,45,46]. The conditions in late winter and spring were nevertheless quite different in 2010 compared to 2011. The Chl *a* values were much higher in 2011 and nanoplankton, bacteria and virus abundances were stable or steadily increasing, although not fluctuating as in 2010. A deeper and more pronounced pycnocline in 2011 than in 2010 resulted in a more stable water column that sustained a longer-lasting bloom in 2011 than in 2010. In summer, fall and early winter, hydrographic conditions for the two years resembled each other, as did Chl *a* level and variation. High abundances of phototropic pico- and nanoeukaryotes and *Synechococcus* (Figure 2) matched peaks in Chl *a* concentration in August 2009, in June, September and October 2010, and in May 2011 (Figure 1). The concentration of Chl *a* in Raunefjorden is, however, largely determined by the abundance of larger phytoplankton forms like diatoms that were not counted in this study [40,44]. On several occasions (e.g., June 2009, June and September 2010, and March–May 2011) the decrease in Chl *a* concentrations may be related to a concurrent deep mixing and exchange of water masses (Figure 1).

### 3.2. Succession of Haptophytes and Co-Occurring DNA Viruses

454 pyrosequencing revealed a high diversity of haptophyte OTUs as well as of algal viruses. The diversity of haptophyte OTUs found in this study was larger than reported in earlier studies using microscopy (summarized in [47]). This demonstrates how high throughput sequencing of amplicon libraries is a powerful tool for detecting haptophyte species not yet morphologically and genetically characterized [48,49]. The level of haptophyte richness measured in Raunefjorden (93 different haptophyte OTUs) was at the same order of magnitude as the level previously found in Oslofjorden (156 haptophyte OTUs), a study for which the same sequencing technology and primers were used [47]. The loss of reads in the filtering process (Table S3) was high but can be explained by the fact that the multiplex identification *tag* was only present on the forward primer, leading to loss of nearly half of the reads.

Both haptophyte and viral communities varied substantially throughout the seasonal cycle. However, we could not distinguish a synchronization between their respective compositions. This may be explained by a complex virus-host relationship, or that our molecular approach was not sensitive enough to capture specific haptophyte viral-host pairs. Most empirical data on phytoplankton diversity resolve the host community diversity on a relatively coarse level, namely with host subgroups or species, typically defined by small subunit ribosomal RNA (SSU rRNA) gene sequences. Viral host-range, however, commonly dwells within strain or sub-species diversity levels [50]. Hence, 18S rRNA gene marker, as used in this and most other studies, might not be sensitive enough to capture the interaction dynamics between the viral sequences here observed and the true host to which they correspond.

Some viral OTUs resembled cultured haptophyte viruses but they were, in most cases, only distantly related. Others were more similar to viruses infecting other host groups. Due to the large diversity of haptophyte viruses and the paucity of isolated viruses infecting this important host group, there is, at present, no molecular approach available that allows us to target these viruses with specificity. Moreover, viral phylogeny does not necessarily reflect host phylogeny. Several algal viruses have been shown to cross host-species borders, and some infect hosts that are only distantly related [25]. Despite these challenges, our viral data did enable detection of successional patterns providing new insight into the interaction between viruses and their hosts. Some viral OTUs were highly abundant only at specific time-points, indicating a boom-bust relationship with their host, a pattern normally described for lytic viruses [11,12,13,15]. Surprisingly though, most of the viral OTUs were persistent indicating coexistence with their hosts, or alternatively an ability to exploit several host species.

Bloom communities normally comprise a few, and often recurrent, species [40,44]. Therefore, we were not surprised to find low haptophyte diversity, dominated by *E. huxleyi*, the common bloom-forming coccolithophores, and Chrysochromulinaceae (OTU001 and OTU004, respectively) in May both years. More to our surprise though, the diverse community of *Phycodneaviridae* and Megaviridae co-occurred with this recurrent, low-diversity haptophyte community, and the observed viral OTUs varied substantially between the two years. One possible explanation may be that genetically different viruses are exploiting the same hosts [25,51,52]. Even more surprising was the absence of the EhV in our flow cytometry (FCM) analysis. Blooms of *E. huxleyi* are frequently succeeded by large increases in this specific virus [53,54,55] which have a FCM characteristics that make them easy to distinguish and recognize [11,56] even without primers that capture their presence at the molecular level.

Our observation that the abundance of pico- and nanoeukaryotes and the diversity of haptophytes peaked in August is in accordance with the general narrative that relatively small forms typically dominate the diverse phytoplankton communities thriving in the temperate, stable, and nutrient-depleted summer water masses. The abundance of larger viruses (V2 and V3), i.e., viruses having a size typical of many dsDNA phytoplankton viruses [40,57], were also at their highest in the summer period. The *Phycodnaviridae* and Megaviridae communities were, however, dominated by a single OTU (OTU010), which was only distantly related to any known viral sequence. Thus, the diverse phytoplankton community in August seems to have sustained a high virus abundance with low diversity. One interpretation of this is that OTU010 represents a generalist virus type, able to infect several different species, a feature known for many viruses infecting members of Prymnesiales [25]. Other interpretations may be that phytoplankton viruses that are not targeted by our primers prevailed [25,32] or that the large viruses are related to other hosts groups.

Several of the haptophytes that we detected in our study are known to be susceptible to characterized algal viruses [24,25,58]. Viruses infecting *P. pouchetii*, *P. globosa, Haptolina ericina and Prymnesium kappa* have previously been isolated from Norwegian coastal waters and/or the North Sea [24,25,58,59], but none of the viral OTUs we found was similar to any of these characterized viruses within 95% aa similarity. Despite the low similarity, five of the OTUs clustered within the Megaviridae group together with several cultured representatives of viruses infecting the orders Prymnesiales and Phaeocystales. As very few algal viruses are cultured, our results may suggest that the diversity within this viral group is large. Alternatively, cultivated viruses may not represent the most abundant viral strains present in natural systems as current procedures for virus isolation [60] entail a strong selective pressure favoring lytic viruses with short replication cycles, a strategy perhaps not very common strategy in nature.

Some haptophyte and viral OTUs were remarkably persistent, considering that the samples were collected at different seasons, interspersed by mixing of the water-masses and changing environmental conditions. Haptophyte OTUs that were present in all samples may represent species that are able to tolerate a wide range of environmental conditions, either as actively dividing cells, or surviving periods of low activity. Most of these persistent OTUs were classified to Prymnesiales (eight OTUs), an order including several mixotrophic species known to survive even when light conditions are too low for photosynthesis [3]. A high degree of preservation and recurrence of virus-genotypes through the years have previously been observed for myovirus-like viruses [46], but this is the first observation for algal viruses. These year-long observations are contrasting and complementary to previous studies demonstrating clear boom and bust patterns for abundant algae and their viruses [15,53,61]. Viral particles are estimated to quickly degrade in seawater (inactivation rates of 5%–20% per h) [62,63,64], and should quickly disappear without co-occurring susceptible and active hosts. Persistent viral OTUs thus indicate either that they propagate and co-exist with a persisting host, or that they are able to infect various species. Virus-host coexistence [19,21,65] is regarded to be a paradox, since most cultured viruses quickly induce resistance in their hosts [21,66] and may be attributed to partial host resistance (strain specificity), low virus infectivity [21], or to chronic infections where only few cells in the host population produce the virus, while the rest grow normally [9,67]. Another possibility is that the persistent viruses have wide host ranges, which would allow them to proliferate on different host species [25]. The ability to infect several species may be especially beneficial in times when the phytoplankton community is very diverse or at low phytoplankton abundance and activity.

This inter-annual study of microbial communities in Raunefjorden is the first to apply high throughput sequencing to study seasonal variation in marine uncultured algal and viral communities. Five “snapshots” of the haptophyte and algal virus (*Phycodnaviridae* and Megaviridae) communities covering one year revealed a large diversity with many uncultured and unknown forms although we identified a stable “core” community of haptophyte and viral OTUs as well. Some abundant viral OTUs showed high relative abundance in several samples indicating virus-host coexistence or wider host-range than what we would expect from the existing isolates. The diversity varied a lot, and low haptophyte diversity in May was accompanied by high algal virus diversity whereas high haptophyte diversity in August co-occurred with low *Phycodnaviridae* and Megaviridae diversity. We suggest that several viruses may exploit the same hosts in the low-diversity spring communities, while a few viruses may be able to exploit several of the haptophytes in the high-diversity community in late summer. Notably, measured virus and host abundance illustrates the importance of viral caused mortality in the diverse late summer community.

## 4. Material and Methods

### 4.1. Sample Collection

Seawater samples were collected from 5 m depth in Raunefjorden (60°16.2′ N, 5°12.5′ E) Western Norway, between May 2009 and May 2011. The sampling interval was, with a few exceptions, 2–4 weeks. Temperature, salinity, density and Chl a fluorescence were determined using a CTD (Conductivity-Temperature-Depth) equipped with an in situ fluorometer (SD204 SAIV, SAIV A/S Environmental Sensors & Systems, Bergen, Norway). A 20 L aliquot of sampled water was filtered by peristaltic pumping through 3.00 µm and then through 0.45 µm pore-sized low-protein-binding filters (145 mm, Durapore, Millipore Corp., Billerica, MA, USA), within 3 h of collection. The filters were cut in two and immediately frozen in liquid N2 and thereafter stored at −80 °C until DNA-extraction for later use in PCR and 454 sequencing of the haptophyte community. Viruses in the 20 L 0.45 µm filtrate were concentrated 400 times (approximately 50 mL) using a tangential flow filtration system equipped with a 100,000 pore size (NMWC) hollow-fiber cartridge (QuixStand, GE Healthcare Bio-Sciences AB, Uppsala, Sweden). Aliquots of these viral concentrates were frozen at -80 °C for later use in PCR and 454 sequencing.

### 4.2. Microbial Abundance Measured by Flow Cytometry (FCM)

Phototrophic pico- and nano- plankton were counted in triplicate by FCM (Becton, Dickinson and Company, BD Biosciences, San Jose, CA, USA), using fresh, unpreserved samples, with the trigger set on red fluorescence and a flow rate giving 50–800 events per sec. Five different populations of phototrophs were defined in FCM-plots based on differences in side scatter, red and orange fluorescence: Synechococcus, picoeukaryotes, nanoeukaryotes, cryptophytes and E. huxleyi ([18,40,56,68], (Table S1)). Most haptophytes fall within the size class pico-and nanoeukaryotes [47,69,70].

Viruses and bacteria were counted in samples preserved with 1% glutaraldehyde (30 min at 4 °C) and snap frozen in liquid N_2_. The samples were thawed, diluted, and stained with 1×SYBR Green I (Invitrogen, Carlsbad, CA, USA 10,000 × conc. in dimethyl sulfoxide (DMSO)) for 10 min at 80 °C [57], immediately before counting in triplicates. Bacteria and three different virus populations were defined based on side scatter properties and green fluorescence: low-, medium- and high-fluorescence viruses (V1, V2 and V3 respectively, [40]). Spearman rank order correlation analyses were calculated in Statistica 12 (StatSoft, Tulsa, OK, USA), to assess the relationship between abundance of different virus and algal groups, as well as their relationship with the measured abiotic factors. Missing values were pairwise deleted.

### 4.3. DNA Extraction, PCR and 454-Pyrosequencing

Five samples, collected on May 25, August 31, and November 30 (2010) and on February 22 and May 31 (2011), were used for targeted 454 pyrosequencing of haptophyte and *Phycodnaviridae/*Megaviridae communities. The samples were chosen based on pulsed field gel electrophoresis (PFGE) analysis to represent different seasonal community stages (Figure S1).

For haptophytes, DNA was extracted from ½ of each 3 µm and 0.45 µm pore-sized filters (representing approximately 10 L of sea water) using DNeasy® Plant Mini kit (Qiagen, Hilden, Germany) according to the manufacturer’s instructions. Initial re-suspension of cells was done by transferring the frozen filters into falcon tubes with 1 ml AP1 buffer (from the DNeasy® kit) and vortexing for 60 s. Extracted DNA from the two different size fractions was subjected to separate PCRs with tagged primers. The V4 region of the 18S rRNA gene (position 640–1060) was amplified using haptophyte-specific primers: 528Flong (5′GCGGTAATTCCAGCTCCAA3′) and PRYM01+7 (5′-GATCAGTGAAAACATCCCTGG-3′) [71]. Each PCR mixture contained 1 µL DNA template, 5 µL Phusion GC buffer (NEB Inc., Ipswich, MA, USA), 0.2 mM each deoxynucleoside triphosphate (dNTP), 400 nM each primer, 0.75 µL DMSO, 0.5 units Phusion® High-Fidelity DNA Polymerase (NEB Inc.), adjusted for a final volume of 25 µL. The cycling parameters were 98 °C for 30 s, and 30 cycles of 98 °C for 10 s, 55 °C for 30 s, and 72 °C for 30 s, with a final extension at 72 °C for 10 min [71].

For viruses, DNA was extracted from 0.5–1 mL of frozen viral concentrate (representing 200–400 mL of sea water). The concentrates were alternately heated to 90 °C and cooled on ice twice for 2 min. Ethylenediaminetetraacetic acid (EDTA, 20 mM) and proteinase K (100 µg/mL) were subsequently added before the samples were incubated for 10 min at 55 °C. Sodium dodecyl sulfate (SDS, final concentration 0.5%) was added and the samples incubated for 1 h at 55 °C. The extracted DNA was then purified with Zymo DNA Clean and Concentrator^TM^ kit (Zymo Research, Irvine, CA, USA) following the manufacturer’s protocols.

A segment of the major capsid protein (MCP) gene was amplified by the primers: MCPforwd (5′-GGY GGY CAR CGY ATT GA-3′) and MCPrev (5′-TGI ARY TGY TCR AYI AGG TA-3′) developed by Larsen et al. [32]. The PCR reactions (25 µL) contained: 0.625 U of HotStarTaq DNA polymerase (Qiagen), 1×PCR buffer, 0.2 mM of each dNTP, 0.5 µM of each of the primers and 1 µL template. The following PCR-program was used: Initial activation at 95 °C (15 min), a touchdown PCR of 20 cycles of denaturation at 94 °C (30 s), annealing at 60 °C and decreasing 0.5 °C per round (30 s) and extension at 72 °C (30 s), followed by 35 cycles with fixed annealing temperature at 45 °C, and a final elongation of 72 °C for 7 min. The PCR products were cleaned (Zymo DNA Clean and Concentrator™ kit) and amplified in new PCR reactions with tagged primers specific to each sample, 25 cycles with annealing at 45 °C, and otherwise as above.

For each haptophyte or viral sample, the products from eight replicate PCR reactions were cleaned, quantified and pooled before sequencing. The DNA amplicons were sent for Roche 454 (GS-FLX Titanium) library sequencing.

Two-directional amplicon sequencing using L chemistry was performed by LGC Genomics GmbH, Berlin, Germany. The following numbers of reads were obtained: haptophytes, 22588 (25 May 2010), 23261 (31 August 2010), 31959 (30 November 2010), 32540 (22 February 2011), 30380 (31 May 2011) (Table S2), viruses, 7909 (25 May 2010), 8195 (31 August 2010), 8558 (30 November 2010), 6791 (22 February 2011), 10353 (31 May 2011) (Table S4).

### 4.4. Sequence Analysis

All reads were filtered using AmpliconNoise [72], with default settings, and further analyzed using Mothur (www.mothur.org) [73] with the commands here provided in italics within brackets. Reads were trimmed (*trim.seqs*) and checked for chimeras by uchime (*chimera.uchime)* with Silva reference sequences for the haptophytes [74].

Prior to clustering, haptophyte reads were aligned to a reference alignment [47] (*align.seqs*) to ensure that the reads aligned in the targeted region, and to enable distance calculation by *dist.seqs*. Based on these distances, reads were clustered de novo into OTUs with 98% similarity (*cluster*), with the average neighbor-algorithm. An OTU definition of 98% nucleotide similarity was applied here in accordance with studies showing this to be a good threshold for delineating different species of most protists, while at the same time accounting for intra-species variation [75]. OTUs of different lengths, but which were otherwise identical, were clustered at 100% similarity by Uclust [76] and the longest sequence of each OTU was picked as representative. OTUs shorter than 250 bp were removed. OTUs that were represented by only a single read (singletons) were excluded from the analysis. Taxonomic classification was performed by MegaBLASTin Geneiuos v. 8.1.6 against the PIP Haptophyte 18S rDNA reference sequence database as described in [42] and available from figshare [77]. Diversity analyses were performed in Mothur (*collect.shared*, *summary.single*). To compare OTU richness between samples, all samples were subsampled to the number of reads in the smallest sample (1320), by the function *rrarefy* in the *vegan* package v. 2.4-1 [78] in R v. 3.3.2 (R Core Team 2016).

As many of the OTUs were found in both size fractions (>3 and 3–0.45 µm), the number of reads in the two size fractions were pooled, and the relative abundance of each OTU in the five samples was determined.

The filtered viral MCP reads were translated into the corresponding amino acid sequence in BioEdit [79]. Alignment of the amino acid reads was done with MAFFT v7) [80], with a gap opening penalty of 2.5, offset value of 0.1, and BLOSUM62 as the amino acid scoring matrix. Insertion/deletion errors were manually corrected. Reads that then did not align in the mid-conserved region (approx. position 100 in the alignment) or contained stop-codons, indicative of sequencing errors, were removed. The remaining reads were trimmed to equal length, i.e., position 117 in the alignment. A protein distance matrix was calculated by PROTDIST v3.5c (©1993 Joseph Felsenstein), and used to cluster the sequences in Mothur [73]. As the large number of PCR-cycles is prone to create artifacts, a 95% amino acid sequence identity threshold was applied [35]. To further decrease the number of spurious reads, only OTUs containing ten or more reads were used in the analysis. Mothur was used for downstream calculations of diversity indices. A representative sequence for each OTU containing more than 50 reads was used for phylogenetic analysis (together representing 84% of the reads). These OTUs were tentatively assigned a phylogenetic affiliation (BLAST-search closest hits) to the Megaviridae or *Phycodnaviridae* family. The tree was constructed, comprising the representative sequences together with reference sequences. Alignment and phylogenetic reconstructions were performed using the function “build” of ETE3 v3.0.0b32 [81] implemented on the GenomeNet, Tree [82] . The tree was constructed using FastTree v2.1.8 with default parameters [83]. Statistical support for the internal branches was calculated by an aLRTtest (SH-like), and through 100 bootstraps. Cluster diagrams were drawn for the haptophyte and virus samples separately. The cluster diagrams were based on Bray Curtis similarities of relative abundance of each virus or haptophyte OTU in the samples. A SIMPROF permutation test was applied to test if the samples could be differentiated at *p* < 0.05 (Primer 6, Primer-E Ltd., Ivybridge, UK).

## Supplementary Materials

The follow supplementary materials can be found online at www.mdpi.com/1999-4915/9/4/84/s1, Table S1: Spearman Rank Order Correlations of the quantitative biological data, including the chl a measurements and the population abundances obtained by flow cytometry. Table S2: Heatmap and OTU table showing the relative abundance and percentage identity to nucleotide blast hits of the haptophyte OTUs in samples from Raunefjorden. Table S3: Results of the 454 sequencing and analysis of the V4-region of 18S rDNA 18S rDNA in haptophytes from Raunefjorden. Figure S1: Schematic representation of the relative abundance of distinct viral populations. Table S4: Result of the 454 sequencing of the viral MCP gene in samples from Raunefjorden. Table S5: Heatmap showing relative abundance of the different viral MCP OTUs in samples from Raunefjorden. Suplementary method and material describing viral diversity explored by pulsed-field gel electrophoresis (PFGE) and method precautions.

## Data Accessibility

The MCP and haptophyte sequences have been submitted to NCBI SRA-database under the bioproject id PRJNA262844.
