# Supplementary material for: Seasonal Dynamics of Haptophytes and dsDNA Algal Viruses Suggest Complex Virus-Host Relationship
Source: Viruses. 2017 Apr 20;9(4):84. doi: 10.3390/v9040084 (PMC5408690; doi:10.3390/v9040084)
Supplement: Supplementary file 1 [file viruses-09-00084-s001.pdf]

Supplementary tables and figures

Table S1

Spearman Rank Order Correlations of the quantitative biological data, including the chl a measurements (i.e. in situ fluorescence) and the population abundances obtained by flowcytometry, were examined using Statistica 12 (StatSoft, Tulsa, OK, USA). Missing data were pairwise deleted. Correlations marked in red and bold are significant at $p < 0.01$.

	Chl a	*Synecho-coccus*	Pico-eukaryotes	Nano-eukaryotes	Crypto-phytes	*E. huxleyi*	Bacteria	V1	V2
Synechococcus	0.022								
Picoeukaryotes	0.252	0.292							
Nanoeukaryotes	**0.634**	**0.525**	0.443						
Cryptophytes	0.374	**0.683**	0.346	**0.812**					
E. huxleyi	**0.551**	0.312	**0.553**	**0.689**	0.463				
Bacteria	0.201	0.473	0.401	**0.490**	0.477	0.317			
V1	**0.519**	**0.563**	0.459	**0.637**	**0.706**	0.392	**0.697**		
V2	0.440	**0.591**	**0.484**	**0.623**	**0.664**	0.303	**0.600**	**0.873**	
V3	0.193	**0.682**	0.321	0.408	**0.527**	0.164	**0.561**	**0.702**	**0.784**

Table S2. Heatmap and OTU table showing the relative abundance and percentage identity to nucleotide blast hits of the haptophyte OTUs (with >1 read) in five samples from Raunefjorden. The alignment coverage was 100 % for all OTUs. The accession numbers shows reference sequences with best match to the OTUs in our study. Color-coding according to relative abundance.

**Group**	**Taxa with best match**	**OTU code**	**% similarity**	**Accession number**	**E-value**	**Max Sequence Length**	**May 2010**	**Aug 2010**	**Nov 2010**	**Feb 2011**	**May 2011**
Isochrysidales	*Emiliania huxleyi /Gephyrocapsa* sp.	OTU001	100	KT861255	0	378	91.48	13.06	39.26	10.08	62.38
Prymnesiaceae	*Dicrateria rotunda*	OTU002	100	KT861304	0	376	0.33	0.04	14.90	23.56	1.66
Phaeocystales	*Phaeocystis pouchetii*	OTU003	100	KR091066	0	375	0.17		1.44	27.88	
Chrysochromulinaceae	*Chrysochromulina* sp.	OTU004	100	HQ868578	0	377	1.92	4.83	0.75	0.61	12.32
Prymnesiaceae	*Prymnesium* sp.	OTU005	100	HQ865286	0	379	0.42	12.08	1.39	1.67	0.20
Chrysochromulinaceae	*Chrysochromulina* sp.	OTU006	100	KJ763084	0	378	0.38	10.23	0.64		0.42
Chrysochromulinaceae	*Chrysochromulina* sp.	OTU007	100	HM581565	0	377		8.59	1.12	0.38	0.24
Chrysochromulinaceae	*Chrysochromulina* sp.	OTU008	100	EF695124	0	379	0.13	5.44	3.53	0.91	0.73
Prymnesiaceae	Clade B3	OTU009	100	JX680412	0	377			1.98	8.56	
Chrysochromulinaceae	*Chrysochromulina* sp.	OTU010	100	HM581628	0	379	0.13	4.43	2.40	0.38	2.80
Prymnesiaceae	*Prymnesium* sp.	OTU011	100	HQ865286	0	376	0.08		7.05	2.95	0.11
Chrysochromulinaceae	*Chrysochromulina* sp.	OTU012	100	EU500076	0	379	0.08	5.22	4.17	0.30	
Prymnesiaceae	*Haptolina* sp.	OTU013	100	AJ246272	0	379	0.63	2.04	0.16	1.67	3.82
Prymnesiaceae	*Prymnesium polylepis*	OTU014	100	FN551246	0	379		2.30		4.39	0.07
Chrysochromulinaceae	*Chrysochromulina* sp.	OTU015	100	HM581635	0	377	0.08	2.30	0.11		2.76
Chrysochromulinaceae	*Chrysochromulina* sp.	OTU016	100	HM561161	0	379	0.42	1.81	1.12	1.44	0.44
Prymnesiophyceae unclassified	Clade F	OTU017	99.7	FN690514	0	379	0.13	2.21	1.71	1.59	0.04
Chrysochromulinaceae	*Chrysochromulina* sp.	OTU018	100	AB180202	0	379	0.08	2.26		0.15	2.72
Prymnesiophyceae unclassified	Clade D	OTU019	100	HM565912	0	379		0.09	5.18		
Chrysochromulinaceae	*Chrysochromulina* sp.	OTU020	100	FJ431487	0	378		5.00			
Coccolithales	*Calyptrosphaera sphaeroidea*	OTU021	100	AM490990	0	376		4.43			
Haptophyta unclassified	Clade HAP5	OTU022	99.5	KF129994	0	378	0.13	0.13	1.50	1.97	0.09
Haptophyta unclassified	Clade HAP4	OTU023	100	EU500064	0	375		3.45			

Chrysochromulinaceae	*Chrysochromulina* sp.	OTU024	100	HQ865016	0	379		0.18	0.27	3.41	
Prymnesiaceae	*Prymnesium* sp.	OTU025	100	JX680402	0	379	0.25	0.04		0.15	4.26
Chrysochromulinaceae	*Chrysochromulina leadbeateri*	OTU026	100	AM491017	0	378	0.08	0.49	0.21	0.68	1.32
Chrysochromulinaceae	*Chrysochromulina* sp.	OTU027	100	HQ868578	0	373		0.09		2.12	0.07
Prymnesiophyceae unclassified	Clade F	OTU028	100	HQ868752	0	379		0.80	0.96	0.15	
Isochrysidales	*Noelaerhabdaceae*	OTU029	98.9	JX680408	0	376	0.96		1.39	0.15	
Phaeocystales	*Phaeocystis globosa*	OTU030	100	JX188372	9.3E-118	225	0.75				0.91
Prymnesiaceae	*Prymnesium* aff. *polylepis*	OTU031	100	AJ004868	0	379	0.08	1.06			0.13
Phaeocystales	*Phaeocystis globosa*	OTU032	100	JX188372	0	374		0.04	0.91	0.53	
Chrysochromulinaceae	*Chrysochromulina* sp.	OTU033	100	KJ762999	0	379			0.48	0.61	0.11
Prymnesiophyceae unclassified	Clade F	OTU034	98.7	FN690514	0	376				1.44	
Chrysochromulinaceae	*Chrysochromulina* sp.	OTU035	100	GU824785	0	379	0.17	0.09	0.64	0.30	
Chrysochromulinaceae	*Chrysochromulina* sp.	OTU036	100	KC488449	0	375	0.21	0.09	0.53		0.26
Chrysochromulinaceae	*Chrysochromulina* sp.	OTU037	100	FJ431495	1.6E-171	322			0.80	0.30	
Prymnesiaceae	*Haptolina* sp.	OTU038	100	KJ763188	0	377	0.17	0.66			
Haptophyta unclassified	Clade HAP2	OTU039	100	FJ537342	0	377					0.44
Syracosphaerales	*Syracosphaerales*	OTU040	100	GU824905	0	376		0.18		0.53	
Phaeocystales	*Phaeocystis cordata*	OTU041	100	FJ431385	0	366		0.71			0.31
Chrysochromulinaceae	*Chrysochromulina* sp.	OTU042	100	JF698782	0	375					0.55
Chrysochromulinaceae	*Chrysochromulina* sp.	OTU043	100	EF695124	2.8E-174	327	0.42				
Chrysochromulinaceae	*Chrysochromulina* sp.	OTU044	100	HM581625	0	376			0.75		
Haptophyta unclassified	Clade HAP3	OTU045	100	HQ868491	0	376		0.22			0.18
Prymnesiophyceae unclassified	*Braarudosphaeraceae*	OTU046	99.7	EU499958	0	374		0.49			
Chrysochromulinaceae	*Chrysochromulina scutellum*	OTU047	100	AJ246274	0	379			0.32	0.15	
Prymnesiaceae	*Prymnesium polylepis*	OTU048	100	FN551246	5.7E-166	312		0.27		0.15	
Chrysochromulinaceae	*Chrysochromulina* sp.	OTU049	100	HM749951	0	378		0.62			
Chrysochromulinaceae	*Chrysochromulina* sp.	OTU050	100	HQ870464	0	376			0.37		0.09
Zygodiscales	*Helicosphaera carteri*	OTU051	100	AM490983	3.2E-153	289		0.40			
Chrysochromulinaceae	*Chrysochromulina* sp.	OTU052	100	EU500076	0	374		0.18	0.11	0.15	
Prymnesiaceae	Clade B4	OTU053	100	GQ863798	0	364			0.37		
Phaeocystales	*Phaeocystis* sp.	OTU054	100	EF695123	0	343		0.49			
Prymnesiaceae	*Prymnesium* sp.	OTU055	100	HM581563	0	379			0.48		0.04

Prymnesiaceae	Clade B3	OTU056	100	JX453461	7.7E-175	328		0.44			
Pavlovales	*Pavlova* sp.	OTU057	100	JF714232	0	374			0.21		0.11
Chrysochromulinaceae	*Chrysochromulina* sp.	OTU058	100	FJ431406	0	379		0.09	0.16		0.15
Chrysochromulinaceae	*Chrysochromulina* sp.	OTU059	100	HQ870181	0	379			0.37		
Pavlovales	*Pavlova* sp.	OTU060	100	AB183598	0	369			0.32		
Phaeocystales	*Phaeocystis globosa*	OTU061	100	JX188372	2.6E-118	226				0.30	
Chrysochromulinaceae	*Chrysochromulina* sp.	OTU062	100	HM581634	0	378		0.09	0.11		
Chrysochromulinaceae	*Chrysochromulina* sp.	OTU063	100	AB180202	0	369		0.35			
Chrysochromulinaceae	*Chrysochromulina* sp.	OTU064	100	KF129692	0	379					0.15
Phaeocystales	*Phaeocystis globosa*	OTU065	100	JX188372	2.6E-118	226		0.27			
Prymnesiaceae	*Prymnesium* aff. *polylepis*	OTU066	100	AJ004868	0	379		0.27			
Prymnesiophyceae unclassified	Clade D	OTU067	100	HM565912	2.1E-170	320		0.22			
Phaeocystales	*Phaeocystis globosa*	OTU068	100	FJ431399	0	374		0.09	0.11		
Chrysochromulinaceae	*Chrysochromulina rotalis*	OTU069	100	AM491025	0	375	0.17				
Prymnesiaceae	Clade B3	OTU070	100	HQ864929	8.8E-149	281			0.21		
Prymnesiaceae	*Prymnesium* sp.	OTU071	100	HM581563	0	379			0.21		
Prymnesiophyceae unclassified	Clade E	OTU072	100	JX680437	0	366			0.21		
Zygodiscales	*Scyphosphaera apsteinii*	OTU073	100	AM490984	0	379		0.18			
Chrysochromulinaceae	*Chrysochromulina* sp.	OTU074	100	HQ869717	0	375				0.23	
Prymnesiaceae	*Prymnesium radiatum*	OTU075	100	FR677016	0	372					0.07
Chrysochromulinaceae	*Chrysochromulina* sp.	OTU076	100	HQ865016	3.2E-153	289		0.22			
Pavlovales	*Pavlova* sp.	OTU077	100	JF714230	2.4E-144	273			0.11		0.04
Phaeocystales	*Phaeocystis globosa*	OTU078	100	JX188372	2.6E-118	226			0.16		
Chrysochromulinaceae	*Chrysochromulina* sp.	OTU079	100	JX680382	0	376			0.16		
Prymnesiaceae	Clade B4	OTU080	100	EU499961	0	367		0.13			
Haptophyta unclassified	Clade HAP2	OTU081	100	FJ537336	0	379			0.11		
Pavlovales	*Diacronema ennorea*	OTU082	100	JF714242	0	375		0.09			
Prymnesiophyceae unclassified	Clade F	OTU083	99.6	FN690514	3.2E-148	284			0.11		
Phaeocystales	*Phaeocystis* sp.	OTU084	100	JX680435	0	376			0.11		
Chrysochromulinaceae	*Chrysochromulina* sp.	OTU085	100	HM581566	0	377				0.15	
Pavlovales	*Pavlova gyrans*	OTU086	100	AF106055	0	373		0.09	0.11		
Chrysochromulinaceae	*Chrysochromulina* sp.	OTU087	100	HQ394067	1.9E-150	284		0.18			

Prymnesiophyceae unclassified	Clade E	OTU088	100	JX680379	0	374	0.17					
Phaeocystales	*Phaeocystis globosa*	OTU089	100	JX188372	9.3E-118	225		0.09				
Prymnesiaceae	Clade B3	OTU090	100	JX680440	0	378		0.09				
Prymnesiaceae	Clade B3	OTU091	100	HM581600	0	363			0.11			
Prymnesiaceae	Clade B3	OTU092	100	JX680412	2.4E-144	273			0.11			
Zygodiscales	*Algirosphaera robusta*	OTU093	100	AM490985	0	373		0.09				

Table S3

Results of the 454 sequencing and analysis of the V4-region of 18S rDNA 18S rDNA in haptophytes from Raunefjorden.

Sample date	Number of sequences	Number of sequences after filtering[1]	Number of OTUs[2]	Number of OTUs[3]	Shannon diversity index[4]
**May 25. 2010**	22588	2395	27	24	0.50
**August 31. 2010**	23261	2259	56	54	2.97
**November 30. 2010**	31959	1872	51	51	2.51
**February 22. 2011**	32540	1320	35	35	2.55
**May 31. 2011**	30380	4535	36	33	1.64

1) Noise and chimera removed in AmpliconNoise.
2) OTUs were defined as having 98 % nucleotide similarity, i.e. reads that had >= 98% identical nucleotides were grouped into one OTU by *de novo* clustering.
3) after subsampling
4) The diversity measures were based on the OTU assignments.

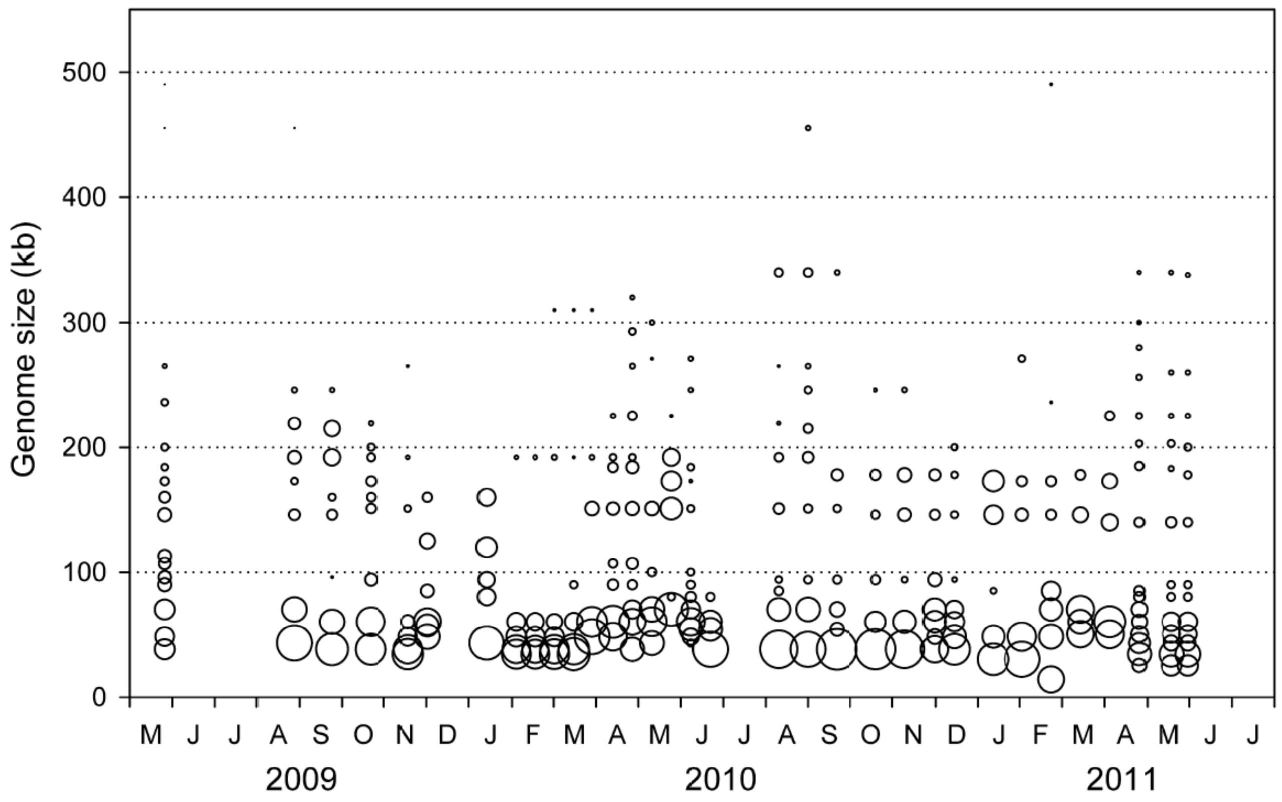

Figure S1. Schematic representation of the relative abundance of distinct viral populations. Populations are defined by genome size determined by PFGE, and based on profiles from two electrophoretic runs for each viral concentrate (see supplementary methods). The relative abundance is normalized to 1 for each date and represented by the area of the circles.

Table S4 . Result of the 454 sequencing of the viral MCP gene from viruses in the *Megaviridae* and *Phycodnaviridae,* in samples from Raunefjorden, and analysis of these sequences.

Sample	Number of sequences	Number of sequences after filtering[1]	Median length	Number of OTUs[2]	Number of OTUs >9 reads	Shannon diversity index[3]
**May 25. 2010**	7909	2044	303	186	41	2.66
**August 31. 2010**	8195	2219	329	117	30	1.45
**November. 30. 2010**	8558	2143	319	143	38	2.14
**February 22. 2011**	6791	1714	323	51	21	1.32
**May 31. 2011**	10353	2479	328	92	30	1.87

1) Noise and chimera removed in AmpliconNoise.
2) OTUs were defined as having 95 % nucleotide similarity.
3) The diversity measures were based on the OTU assignments.

Table S5. Heatmap showing the relative abundance of the different OTUs containing 10 and more reads (i.e. 86-96% of all reads) in five samples from Raunefjorden. Sequences were generated from 454 sequencing targeting *Phycodnaviridae* and Megaviridae in Raunefjorden. Relative abundance is shown as percentage of the total sequences in each sample. OUT are sorted after phylogenetic placement in the two families *Phycodnaviridae* or Megaviridae. Color-coding according to relative abundance.

Group	OTU #	May_10	Aug_10	Nov_10	Feb_11	May_11
Phycodnaviridae	OTU124	3.1				1.3
Phycodnaviridae	OTU009	0.44	0.18	2.8	0.06	0.08
Phycodnaviridae	OTU027	1.1	0.68	0.8		0.04
Phycodnaviridae	OTU329	2				
Phycodnaviridae	OTU058	0.3		1.1		
Phycodnaviridae	OTU043			0.47		
Phycodnaviridae	OTU002	0.84	12	41	7.7	38
Phycodnaviridae	OTU001	3.9	8.5	4.3	2.3	20
Phycodnaviridae	OTU003	0.34	2	8.6	1.7	18
Phycodnaviridae	OTU373	13				
Phycodnaviridae	OTU007		0.09	0.28	0.29	2
Phycodnaviridae	OTU005	0.25	0.77	0.38		0.65
Phycodnaviridae	OTU069			0.05		0.97
Phycodnaviridae	OTU115	0.05				0.36
Phycodnaviridae	OTU013		0.23	0.09		0.08
Phycodnaviridae	OTU016	6.9	1	0.89		0.4
Phycodnaviridae	OTU113	7.4				
Phycodnaviridae	OTU012	1.3	0.23	0.14		0.04
Phycodnaviridae	OTU075		0.46	0.47		

Phycodnaviridae	OTU110	0.84				
Megaviridae	OTU010		58	0.05		0.04
Megaviridae	OTU018	3.5	0.32	2.5	0.64	
Megaviridae	OTU064			2.4	0.12	1.2
Megaviridae	OTU046	1.4	0.32	0.05		
Megaviridae	OTU031		0.91			
Megaviridae	OTU077		0.91			
Megaviridae	OTU055		0.09	0.38	0.29	
Megaviridae	OTU004	2.5	0.05		2.6	2.1
Megaviridae	OTU021	0.1	0.05		0.06	4.3
Megaviridae	OTU011	1.4			1.2	0.24
Megaviridae	OTU114	0.94				
Megaviridae	OTU131			0.7		
Megaviridae	OTU118	0.54				0.04
Megaviridae	OTU033	0.05	0.41	0.05		
Megaviridae	OTU141			0.47		
Megaviridae	OTU008	1.6	0.32	12	65	3.1
Megaviridae	OTU006	25		0.05	0.06	0.08
Megaviridae	OTU040	0.39		0.47	9.9	0.04
Megaviridae	OTU037	0.84		4.4	0.18	
Megaviridae	OTU068		0.09	2.4	1.2	
Megaviridae	OTU044		0.05		1.9	0.04
Megaviridae	OTU020	0.05	0.64	0.28		0.97
Megaviridae	OTU052	0.1				1.1
Megaviridae	OTU048		1.1	0.05		

Megaviridae	OTU143	1.1		0.05		
Megaviridae	OTU025			0.56	0.58	
Megaviridae	OTU014	0.05	0.23	0.23		0.49
Megaviridae	OTU188	0.74				
Megaviridae	OTU101	0.54				0.04
Megaviridae	OTU023		0.27	0.19	0.06	
Megaviridae	OTU136			0.52		
Megaviridae	OTU167	2				
Megaviridae	OTU019	0.39	0.05	0.42		
Megaviridae	OTU184			0.85		
Megaviridae	OTU015	0.1		0.42	0.23	0.04
Megaviridae	OTU062	0.1		0.61		
Megaviridae	OTU340		0.68			
Megaviridae	OTU017	0.54				0.08
Megaviridae	OTU083		0.46	0.09		
Megaviridae	OTU326	0.49				
Megaviridae	OTU106	0.1				0.32

Supplementary methods and material

Viral diversity explored by pulsed-field gel electrophoresis (PFGE)

PFGE was used to assess richness of dsDNA virus genomes of sizes between ~20 and 1000 kbp, as described earlier [1,2]. Within one day after the sampling, 35 mL of each viral concentrate, originating from 7-14 L of sea water, was pelleted by ultracentrifugation for 2 h at 25000 rpm (Beckman Coulter Optima L90K ultracentrifuge and SW32Ti rotor). Viral pellets were resuspended in 200 µl SM-buffer (0.1 M NaCl, 8 mM $MgSO_4 \times 7\ H_2O$, 50 mM Tris-HCl, 0.005 % Glycerin) [2] and mixed 1:1 with agarose (1.5 %, InCert, FMC Bioproducts) to make PFGE plugs. Lysis of the viral particles was performed in fresh lysis buffer (1 mg/mL proteinase K, 1 % SDS, 250 mM EDTA, pH 8.0) o. n. at 30 °C. The plugs were then washed three times in TE 10:1 (10 mM Tris-Base, 1 mM EDTA, pH 8.0) and kept in TE 20:50 (20 mM Tris Base 50 mM EDTA, pH 8.0) at 4 °C until use. The agarose plugs were run on a 1 % agarose gels (Seakem GTG agarose) together with DNA size standards (CHEF DNA size standard lambda ladder and 5 kb ladder, DNA size standard, BIO-RAD). The gels were run in 0.5×TBE buffer (1×TBE: 89 mM Tris-base, 89 mM boric acid, 2 mM EDTA, pH 8.0), at 6V for 22 h at 14 °C, with pulses of 1-8 s (separating genomes from 5-200 kbp) or 8-30 s (separating genomes from 50 -500 kbp). Gels were stained in 1×SYBR Green I (Invitrogen) for 45 min and washed for 15 min in 1×TBE. DNA bands were visualized in a Bio-Rad ChemiDoc system and analyzed using the ImageLab3 software (Bio-Rad).

Method precautions

Several precautions to minimize the biases in DNA-extraction, PCR and sequencing were applied in this study, recognizing the biases associated with high-throughput sequencing. The

output data is not quantitative [3], but might give information of the abundance of an OTU relative to other specific organisms. Due to careful quality-control of the sequences, we feel confident to draw firm conclusions about species or genotypes present, and the phylogenetic affiliation of the haptophytes.

Supplementary references

1. Sandaa, R.-A.; Short, S. M.; Schroeder, D. C., Fingerprinting aquatic virus communities. In *Manual of Aquatic Viral Ecology*, Wilhelm S.W.; Weinbauer M.G.; C.A., S., Eds. ASLO: **2010**; pp 9–18.

2.Wommack, K. E.; Ravel, J.; Hill, R. T.; Chun, J. S.; Colwell, R. R., Population dynamics of Chesapeake bay virioplankton: Total-community analysis by pulsed-field gel electrophoresis. *Appl. Environ. Microbiol.* **1999,** 65, (1), 231-240.

3. Egge, E.S.; Bittner, L.; Andersen, T.; Audic, S.;, de Vargas, C.; Edvardsen, B. 454 Pyrosequencing to Describe Microbial Eukaryotic Community Composition, Diversity and Relative Abundance: A Test for Marine Haptophytes. *PLoS ONE* **2013,** *8***:** e74371.
